# *Methanocella conradii* sp. nov., a Thermophilic, Obligate Hydrogenotrophic Methanogen, Isolated from Chinese Rice Field Soil

**DOI:** 10.1371/journal.pone.0035279

**Published:** 2012-04-17

**Authors:** Zhe Lü, Yahai Lu

**Affiliations:** College of Resources and Environmental Sciences, China Agricultural University, Beijing, China; The University of Hong Kong, China

## Abstract

**Background:**

*Methanocellales* contributes significantly to anthropogenic methane emissions that cause global warming, but few pure cultures for *Methanocellales* are available to permit subsequent laboratory studies (physiology, biochemistry, etc.).

**Methodology/Principal Findings:**

By combining anaerobic culture and molecular techniques, a novel thermophilic methanogen, strain HZ254^T^ was isolated from a Chinese rice field soil located in Hangzhou, China. The phylogenetic analyses of both the 16S rRNA gene and *mcrA* gene (encoding the *α* subunit of methyl-coenzyme M reductase) confirmed its affiliation with *Methanocellales*, and *Methanocella paludicola* SANAE^T^ was the most closely related species. Cells were non-motile rods, albeit with a flagellum, 1.4–2.8 µm long and by 0.2–0.3 µm in width. They grew at 37–60°C (optimally at 55°C) and salinity of 0–5 g NaCl l^−1^ (optimally at 0–1 g NaCl l^−1^). The pH range for growth was 6.4–7.2 (optimum 6.8). Under the optimum growth condition, the doubling time was 6.5–7.8 h, which is the shortest ever observed in *Methanocellales*. Strain HZ254^T^ utilized H_2_/CO_2_ but not formate for growth and methane production. The DNA G+C content of this organism was 52.7 mol%. The sequence identities of 16S rRNA gene and *mcrA* gene between strain HZ254^T^ and SANAE^T^ were 95.0 and 87.5% respectively, and the genome based Average Nucleotide Identity value between them was 74.8%. These two strains differed in phenotypic features with regard to substrate utilization, possession of a flagellum, doubling time (under optimal conditions), NaCl and temperature ranges. Taking account of the phenotypic and phylogenetic characteristics, we propose strain HZ254^T^ as a representative of a novel species, *Methanocella conradii* sp. nov. The type strain is HZ254^T^ ( = CGMCC 1.5162^T^ = JCM 17849^T^ = DSM 24694^T^).

**Conclusions/Significance:**

Strain HZ254^T^ could potentially serve as an excellent laboratory model for studying *Methanocellales* due to its fast growth and consistent cultivability.

## Introduction

The order *Methanocellales*, previously recognized as uncultured archaeal group Rice Cluster I (RC-I), plays a key role in methane production from rice field soils [1], [2], [3]. Members of *Methanocellales* are widely distributed in various environments [1], which further strengthens their roles in global carbon cycling, especially in those microaerophilic environments [4]. However, despite the early detection by molecular techniques in the late 1990s [5], pure cultures of RC-I were not obtained until recently due to their slow growth and fastidious culture conditions [6], [7], [8]. The first axenic culture, a mesophilic hydrogenotrophic methanogen *Methanocella paludicola* strain SANAE^T^, was isolated under low hydrogen concentrations (<30 Pa) from a Japanese rice field soil [9], [10]. The second isolate, a thermophilic, hydrogenotrophic methanogen *Methanocella arvoryzae* strain MRE50^T^, was purified recently from an enrichment culture which had been established since 2000 [7], [11].

Many ecological questions of importance that are difficult to solve by culture-independent methods remain to be answered by pure culture studies of *Methanocellales*. For example, (1) why are members of *Methanocellales* more active under low hydrogen partial pressures prevailing in their natural habitats in comparison with other methanogens [1], [12]? (2) Why do they become predominant at moderate high temperatures while their natural habitats are often mesophilic [13], [14]? (3) How are they able to regulate the expression and translation of their antioxidant machinery thus allowing a presumable adaptation to microaerophilic and even oxic environments [2], [4], [15]? However, despite a successful yet difficult isolation of strain SANAE^T^ and MRE50^T^ and their ecological significance, to the best of our knowledge, no subsequent cultivation of them has been reported. In fact, many workers that we know including ourselves have failed to cultivate those strains. Therefore, the available *Methanocellales* strains remain difficult to cultivate, and more isolates particularly fast-growing ones are needed to push the studies of *Methanocellales* forward.

Here we report the isolation, physiology and phylogeny of the fastest-growing strain of the order *Methanocellales*, and propose a new species, *Methanocella conradii* sp. nov. Its consistency of cultivability is presented as well.

## Materials and Methods

### Samples and medium

Rice field soil was collected in 2006 from an experimental farm at the China National Rice Research Institute in Hangzhou, China (30°04′37″N, 119°54′37″E). No specific permits were required for the described field studies, as the location is not privately-owned or protected and the field studies did not involve endangered or protected species. Soil sample characteristics and storage were described previously [14]. Soil samples were first inoculated into distilled water (10 g soil plus 10 ml water) for a pre-incubation of up to 100 days. Approximately 5 g pre-incubated soil slurries were inoculated into a modified basal medium [16], which was prepared by adding 3 ml of trace element solution, 1 ml of tungstate solution, 2 ml of 0.5 M Na_2_S solution, 1 ml of 1 M bicarbonate solution, and 1 ml each of the three different vitamin solutions into 1 liter of freshwater medium. The freshwater medium contained 0.4 g MgCl_2_.6H_2_O, 0.1 g CaCl_2_.2H_2_O, 0.1 g NH_4_Cl, 0.2 g KH_2_PO_4_, 0.5 g KCl, 0.3 g L-cysteine-HCl.2H_2_O, and 0.0005 g resazurin per liter of distilled water. The freshwater medium was autoclaved and cooled under N_2_ before supplementing with any solutions. The trace element solution was prepared by dissolving 2.000 g FeCl_2_.4H_2_O, 0.070 g ZnCl_2_, 0.100 g MnCl_2_.4H_2_O, 0.060 g H_3_BO_3_, 0.190 g CoCl_2_.6H_2_O, 0.002 g CuCl_2_.2H_2_O, 0.024 g NiCl_2_.6H_2_O, and 0.036 g Na_2_MoO_4_.2H_2_O into 50 ml of 2 M HCl, then diluting to 1 liter with distilled water. The tungstate solution contained 0.4 g NaOH and 0.007 g Na_2_WO_4_.2H_2_O per liter of distilled water. Vitamin solution 1 contained 0.04 g 4-aminobenzoic acid, 0.01 g D(+)-biotin, 0.01 g DL-α-lipoic acid, 0.1 g calcium-D(+)-pantothenate, 0.1 g vitamin B_6_, 0.03 g folic acid, 0.05 g nicotinic acid and 0.05 g vitamin B_2_. The vitamins were dissolved into 1 liter of 50 mM Na-phosphate buffer (pH 7.1). Vitamin solution 2 was consisted of 0.01 g thiamine hydrochloride dissolved in 1 liter of 25 mM Na-phosphate buffer (pH 3.4). Vitamin solution 3 contained 0.05 g vitamin B12 dissolved in 1 liter of distilled water. The vitamin solutions and Na_2_S solution were filter-sterilized (0.2 µm pore size) with N_2_ in the headspace. The bicarbonate solution was autoclaved and saturated with CO_2_. All other solutions were autoclaved with N_2_ in the headspace.

### Enrichments and cultivations

All enrichments and cultivations were performed at 50°C in 100 ml serum bottles under ca. 150 kPa H_2_/CO_2_ (80/20, v/v) except that the pre-incubation was under an atmosphere of N_2_. In the initial several enrichments, 1 g l^−1^ NaCl was also included in the medium. Unless otherwise mentioned, 1 mM acetate and 0.02% yeast extract was normally included in the standard medium except during the initial several enrichments. Isolation using roll tubes or deep agar was described previously [16], [17]. After isolation, all incubations were in liquid medium at pH of 6.8 at 55°C, corresponding to a pH of approximately 7.2 at 25°C, under an atmosphere of H_2_/CO_2_ (80/20 [v/v]) or N_2_/CO_2_ (80/20 [v/v]) without shaking, unless otherwise noted. Substrate utilization and antibiotic (200 mg l^−1^) and SDS (0, 0.1, 0.5, 1.0, 1.5, 2.0%) susceptibility were performed in 17 ml tubes containing 5 ml medium. Tests for growth temperature, pH and salinity range were carried out in 50 ml vials containing 25 ml medium at 25 to 70°C, pH 6.0 to 8.0, and 0 to 10 g NaCl l^−1^. The pH was additionally buffered with 10 mM Bicine, which has a pKa of 7.78 at 55°C [18], and pH values were adjusted at 55°C by adding HCl or NaOH solutions. Growth and substrate utilization were monitored by following the concentration of methane using a gas chromatograph GC-7890A with a thermal conductivity detector (Agilent Technologies). All measurements were performed at least in duplicate, and all incubations were terminated after 1 month unless otherwise mentioned.

### Light and electron microcopy

Cell morphology and motility were examined with a phase contrast microscope (Olympus CX41) equipped with a CCD camera (Canon 450D). Phase-contrast micrographs were taken by preparing agar-coated slides for exponential-phase cultures. Colony morphology and fluorescence were visualized by a light microscope (Olympus CX51) equipped with a CDD camera (Olympus DP71) and a fluorescence illumination system (X-cite 120). Cells of strain HZ254^T^ for thin-section electron microscopy were fixed with 2.5% glutaraldehyde overnight, washed with phosphate buffer (pH 7.2, 0.1 M), and post-fixed in 1% osmium tetroxide for 1 h. The fixed cells were washed again with phosphate buffer (pH 7.2, 0.1 M), dehydrated in the serial steps of acetone (30, 50, 70, 80, 90 and 100%), and embedded in Spurr low-viscosity resin. Thin-sections of the cells were made with an ultramicrotome (LEICAUC6i) and stained with uranyl acetate and lead citrate. Transmission electron micrographs were taken by JEM-123O.

### PCR amplification, sequencing and phylogenetic analysis

DNA extraction, PCR amplification, terminal restriction fragment length polymorphism (T-RFLP) analysis, cloning and sequencing were performed as previously reported [14]. Sequences were either aligned with RDP's aligner tool using the Ribosomal Database Project (RDP 10) [19] for 16S rRNA gene or with the Mega 4.0.2 software package [20] for the deduced McrA amino acid. All sequence alignments were analyzed with the Mega 4.0.2 software package [20]. Distances were calculated using the Jukes-Cantor correction. For phylogenetic analysis, the near full length 16S rRNA gene was amplified using the universal archaeal primer pair Arc21f/1492r [21]. The *mcrA* gene was amplified with MCRf/MCRr [22]. Phylogenetic trees were produced using the neighbor-joining and maximum-parsimony methods by bootstrap re-sampling analysis with 1000 replicates. 16S rRNA gene and *mcrA* gene sequences of strain HZ254^T^ have been deposited in GenBank under the accession numbers of JN048683 and JN081865, respectively.

### Average Nucleotide Identity

Complete genomes of HZ254^T^ (accession number: CP003243) [23], SANAE^T^ (accession number: AP011532) [24] and MRE50^T^ (accession number: AM114193) [2] were used to calculate the ANI (Average Nucleotide Identity) values by the Blast-based method [25] with the Jspecies package [26]. Please note, although the genome for MRE50^T^ was constructed as a complete metagenome from an enrichment before its isolation [2], the fact that MRE50^T^ was the only archaeal member in that enrichment includes that its genome was well represented by the metagenome [6], [11].

## Results and Discussion

### Enrichment and isolation

**Figure 1:**
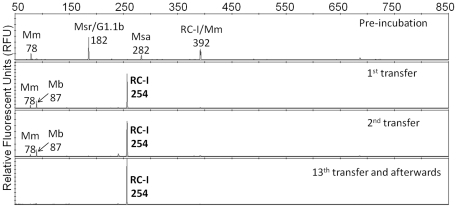
T-RFLP patterns based on 16S rRNA genes for enrichment cultures of strain HZ254^T^ along with successive transfers. The analysis was performed using Ar109f/915r primer set and *TaqI* restriction enzymes [14]. T-RFLP fingerprints were normalized to a total of 100 relative fluorescence units (RFU), and T-RF peaks with RFU less than 1 were discarded. The 254-bp T-RF was affiliated with *Methanocellales* (RC-I) as determined by cloning and sequencing of 16S rRNA genes, and the T-RF length calculated from the sequence was actually 258-bp (data not shown). All other T-RF peaks could be assigned correspondingly to *Methanomicrobiales* (Mm), *Methanobacteriales* (Mb), *Methanosarcinaceae* (Msr)/Crenarchaeotal group 1.1b (G1.1b), *Methanosaetaceae* (Msa) and RC-I/*Methanomicrobiales* (Mm), according to our previous studies in the same soil [14], [34], [35], [36], respectively. The pre-incubation samples were sampled after 24 hours of incubation, and all other samples were sampled after that methane production ceased and/or hydrogen could not be detected in the headspace. After the 13^th^ transfer, the archaeal community was still frequently monitored by T-RFLP analysis along with subsequent transfers, but the 254-bp was always the sole T-RF product.

Enrichment of strain HZ254^T^ was directed by both gas and molecular analyses. Measurement of hydrogen consumption and methane formation and T-RFLP analysis based on 16S rRNA genes were performed frequently to monitor the methanogenic activity and the structure of the archaeal and bacterial communities in the enrichment cultures. Cloning and sequencing of the 16S rRNA genes were also conducted occasionally to determine the identity of the predominant archaeal and bacterial groups. Enrichment cultures with neither RC-I as the predominant archaeal group nor significant methanogenic activity were abandoned. The T-RF patterns for the archaeal community along with the successive transfers of the successful enrichments for strain HZ254^T^ are shown in Figure 1. The figure demonstrates that RC-I quickly predominated after just the first transfer from pre-incubated soil slurries, and it exclusively represented the sole archaeal member after at most 13 successive transfers over 338 days, albeit a diverse archaeal community was present during the initial pre-incubation. Therefore, besides the low hydrogen method [9], our results demonstrate that moderate high temperature remains an effective strategy for enrichment of RC-I methanogens, which is consistent with previous studies that RC-I became predominant upon incubation at 45 to 50°C [13], [14]. Nevertheless, novel methods are needed to increase the cultivability of RC-I. The combination of the two already effective methods (i.e. by inoculating thermophilic propionate- or acetate-degrading syntrophs into samples incubating at 45 to 55°C with propionate or acetate as substrates) would be an approach worth trying, because it may provide a more selective environment for RC-I. Indeed, in both the Chinese and Italian rice field soils, the predominance of RC-I under syntrophic acetate-degrading conditions was observed at 50°C [27], [28].

Isolation was carried out after the establishment of a stable enrichment culture with RC-I as the sole archaeal group. Deep agar and roll tubes were prepared in an attempt to isolate RC-I colonies. However, colonies formed under standard conditions belonged to bacteria instead of RC-I as screened by 16S rRNA gene sequencing. Therefore, various efforts were made to grow colonies. Firstly, cofactors (e.g. acetate, yeast extract, soil extract, sludge extract and coenzyme M) were supplemented in the medium both individually and in combination. Secondly, agar concentrations of 1.50%, 1.75% and 2.00% were tried. Lastly, antibiotics were included occasionally to eliminate bacteria. The roll tube medium that worked contained 1.50% agar supplemented with 0.05% yeast extract and tryptone and 1 mM acetate. Under these conditions, blue fluorescent colonies of RC-I appeared in several roll tubes after 5 months of incubation, as determined by 16S rRNA gene sequencing. The colonies were picked with Pasteur pipette and further purified by serial dilution in liquid medium supplemented with 200 mg l^−1^ kanamycin.

The purity of the culture was confirmed by four criteria: (1) the failure to grow in anoxic PYG medium; (2) the failure to detect bacterial 16S rRNA gene using the universal bacterial primer pair 27f (5′-AGAGTTTGA TCMTGGCTCAG-3′) and 907r (5′-CCGTCAATTCMTTTRAGTTT-3′); (3) a homogenous cell morphology by phase contrast microscopy; (4) homogenous 16S rRNA gene sequences of 27 clones (pair-wise sequence similarity >99.9%) obtained using the universal archaeal primer pair Arc21f/1492r. All results indicated that the HZ254^T^ culture was axenic.

### Morphology

**Figure 2:**
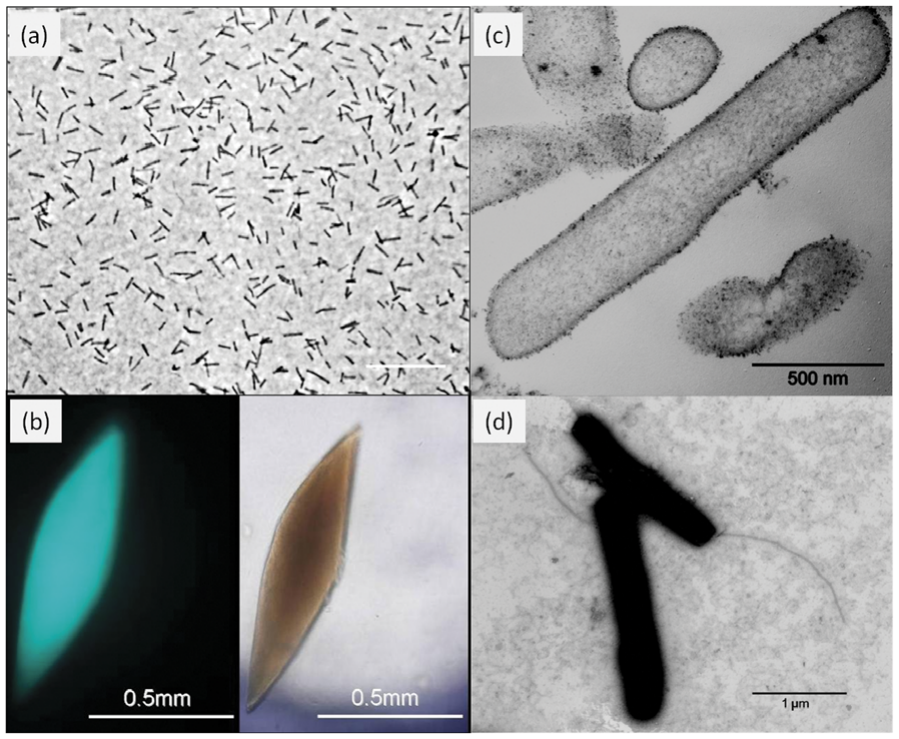
Photomicrographs of strain HZ254^T^. (**a**) Phase contrast micrograph; (**b**) fluorescence (left) and bright field (right) micrographs of the lens-shaped colony in the same field of view; transmission electron micrograph of (**c**) a thin section and of (**d**) negatively stained cells with flagellum; Bars, 10 µm (**a**); 0.5 mm (**b**); 500 nm (**c**); 1 µm (**d**).

Colonies of strain HZ254^T^ were nearly lens-shaped. Both the cells (not shown) and colonies autofluorescenced when excited at 420 nm under an epifluorescence microscope (Figure 2b), which is a characteristic feature of methanogens. Single cells were rod-shaped, 1.4–2.8 µm long and 0.2–0.3 µm wide (Figure 2a). No specific intracytoplasmic structures (intracytoplasmic membranes, inclusion bodies, etc) were found in the cells (Figure 2c). A flagellum was observed after negative staining of the cells (Figure 2d), which was consistent with the presence of a *fla* gene cluster encoding the flagellum in its genome [23]. Therefore, strain HZ254^T^ is probably motile. However, motility was not observed under our laboratory conditions. Further analyses would be needed to test its motility under different conditions.

### Growth requirements

Strain HZ254^T^ utilized H_2_/CO_2_ for growth and methane production but not the following tested substrates: 40 mM formate; 20 mM acetate, propionate, lactate or pyruvate; 10 mM methanol, ethanol, 1-propanol, 2-propanol, 1-butanol, 2-butanol or cyclopentanol; and 10 mM methylamine or trimethylamine. Acetate (1 mM) was required as a carbon source for growth. Yeast extract (0.02%) stimulated growth but was not required.

### Growth parameters, antibiotic and SDS sensitivity

Growth for strain HZ254^T^ was observed at 37 to 60°C with an optimum at 55°C (Figure S1a). The upper limit of methanogenesis observed in rice soil samples so far is below 60°C [13], thus our results suggest a potentially new upper boundary (60°C) for methane emission from rice field soils. However, further studies using environmental samples are still needed to confirm this new boundary. The pH range for growth was between pH 6.4 and 7.2, with an optimum around pH 6.8 (Figure S1b). Although a small amount of methane (partial pressure up to 1 kPa) was produced at pH 7.4 over the initial 5 days of incubation, the amount of methane did not increase during a prolonged incubation up to 50 days. The pH remained nearly constant during incubation for most of the treatments. However, at pH 6.8 and 7.2, a slight increase of 0.4 units was observed at the end of the incubation. The strain grew at NaCl concentrations ranging from 0 to 5 g l^−1^, the optimum growth occurred at 0 to 1 g l^−1^ (Figure S1c). Under optimal conditions (pH 6.8, 55°C, without NaCl), the doubling time calculated from the methane production rate was 6.5 to 7.8 hours (Figure S1d), which was the shortest so far observed in *Methanocellales* (Table 1). The maximum specific growth rates calculated from both model fitting and linear regression analyses were consistently around 0.1 h^−1^ (Figure S1a and S1d). Strain HZ254^T^ could tolerate ampicillin, penicillin-G and kanamycin, but not apramycin, neomycin, rifampicin and chloramphenicol. Cells lysed in 0.5% but not under <0.1% of SDS, and intact cells were hardly seen at 1–2% of SDS when observed by a phase contrast microscope.

**Table 1:** Comparative characteristics of strain HZ254^T^ and *Methanocella paludicola* SANAE^T^ and *Methanocella arvoryzae* MRE50^T^.

Characteristics	HZ254	MRE50	SANAE
Cell morphology	rod	rod, coccoid	rod, coccoid
Cell width (µm)	0.2–0.3	0.4–0.7	0.3–0.6
Cell length (µm)	1.4–2.8	1.3–2.8	1.8–2.4
GC content (mol %)	52.7*	54.6 (56.7)	54.9 (56.6)
Flagellum	+	+	−
Temperature range (optimum) (°C)	37–60 (55)	37–55 (45)	25–40 (35–37)
pH range (optimum)	6.4–7.2 (6.8)†	6.0–7.8 (7)	6.5–7.8 (7)
NaCl range (optimum) (g l^−1^)	0–5 (0–1)	0–20 (0–2)	0–1 (0)
Doubling time (h)	6.5–7.8	8.0	100.8
**ANI values (%)**			
Versus HZ254	N.A.	69.6	74.8
Versus MRE50	69.4	N.A.	70.5
Versus SANAE	74.8	70.6	N.A.
**Substrate utilization**			
H_2_/CO_2_	+	+	+
Formate	−	+	+
Acetate	−	−	−
Methanol or Methylamines	−	−	−
Secondary alcohols	−	−	−
**Tolerance for antibiotics**			
Rifampicin	−	+	−

Data for strain HZ254^T^ is from this study, and strain SANAE^T^ and MRE50^T^ were retrieved from Sakai *et al.*, 2008 and 2010.

* The data in parentheses were determined by HPLC, other data were taken from genome information [2], [23], [24].

† pH for HZ254^T^ and other strains were determined at 55°C and 25°C, respectively. Abbreviations, −, negative; +, positive; N.A., not applicable.

### Consistency of cultivability

Because of the probable difficulty in cultivation of available *Methanocellales* species, special focus was paid to assess the cultivable consistency of strain HZ254^T^. An excellent consistency for cultivating strain HZ254^T^ was judged by three empirical standards: (1) the strain could well survive through successive transfers (seven transfers over more than two years, Table S1); (2) the strain was able to recover from rather long time of storage at 4°C (the maximum storage time allowing recovery was 502 days so far, Table S1); (3) multiple persons within our laboratory could successfully handle the cultivation of the strain. Therefore, strain HZ254^T^ could serve an excellent starting material for laboratory studies of *Methanocellales*.

### GC%, phylogenetic and ANI analyses

**Figure 3:**
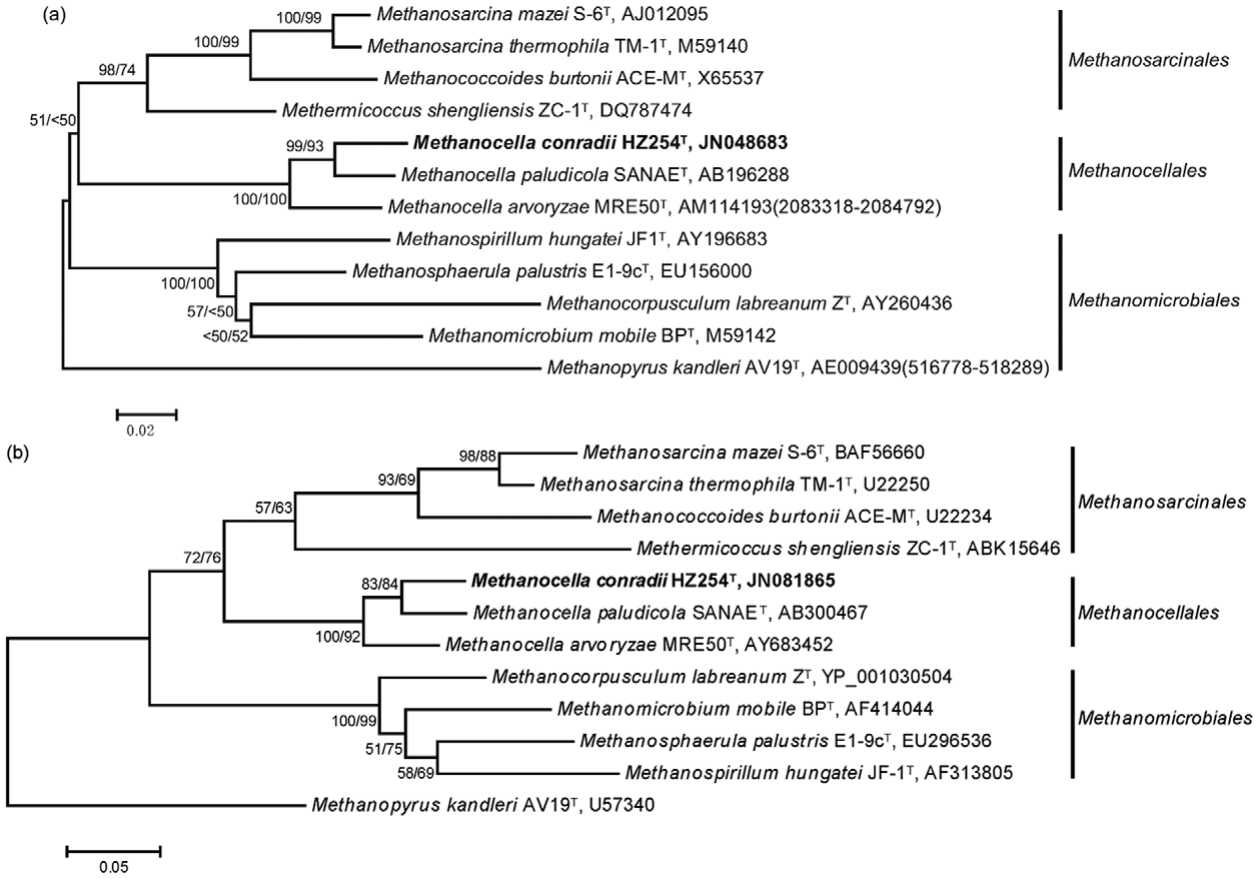
Phylogeny of strain HZ254^T^ based on (a) 16S rRNA gene and (b) deduced McrA amino acid sequences. The trees were constructed using neighbor-joining method. The McrA tree is based on 155 deduced amino acid positions and Poisson correction. The sequences of *Methanopyrus kandleri* AV19^T^, (AE009439; 516778–518289) and (U57340) were used as out groups for rooting the 16S rRNA gene and McrA trees, respectively. The accession number of each reference sequence is shown after the strain name. The coordinates of the sequence were indicated in parenthesis, if it was taken from the complete genome sequence. Bootstrap support (>50% indicated only) was obtained from neighbor-joining (first value) and maximum-parsimony (second value) based on 1000 replicates. The bar represents the number of changes per sequence position.

The DNA G+C content of strain HZ254^T^, as determined by genome sequencing, was 52.7 mol% [23]. Strain HZ254^T^ is affiliated with the order *Methanocellales*, as revealed by the phylogenetic analyses based on the 16S rRNA and *mcrA* genes (Figure 3 and see Figure S2 and S3 for the detailed alignments). The closest relative of strain HZ254^T^ was *M. paludicola* SANAE^T^, having gene sequence identities of 95.0% for 16S rRNA gene and 87.5% (nucleotide level) or 94.1% (amino acid level) for the *mcrA* gene. The corresponding sequence identities between strain HZ254^T^ and *M. arvoryzae* MRE50^T^ were 92.4–92.5% and 86.5 or 92.0% respectively, and the slight variation for the former values is due to the presence of two slightly different copies of 16S rRNA genes within the genome of strain MRE50^T^. Moreover, the calculated ANI values among the three strains of *Methanocella* based on their complete genome sequences were between 69.4 to 74.8%.

### Taxonomic conclusions

The collective traits of strain HZ254^T^ with regard to its physiology and phylogeny support it as a member of the order *Methanocellales*. It shares common phenotypic features with the other two strains (*M*. *arvoryzae* MRE50^T^ and *M. paludicola* SANAE^T^) of *Methanocellales*, such as the rod-shaped morphology, the growth via hydrogenotrophic methanogenesis and the requirement of acetate as a carbon source. However, they differ in formate utilization, possession of a flagellum, antibiotic susceptibility, temperature range, pH range and salinity range. In addition, the ANI values further distinguish the three strains on the species level, given that they are far below 95 to 96% which is the suggested boundary for species delineation [25]. The comparative characteristics of strain HZ254^T^, MRE50^T^ and SANAE^T^ are listed in Table 1. Interestingly, strain HZ254^T^ seems to be closer to MRE50^T^ than SANAE^T^ in major phenotypic traits including temperature range, possession of a flagellum and salinity range, albeit it more resembles SANAE^T^ in regard of 16S rRNA and *mcrA* genes and ANI.

The 16S rRNA gene sequence divergence of 5% between HZ254^T^ and SANAE^T^ implies that strain HZ254^T^ could potentially represent a new genus within *Methanocellales*, given that it is generally considered that a 5 to 7% divergence of 16S rRNA gene sequence is sufficient to delineate different genera [29]. However, the knowledge regarding the physiology of *Methanocellales* is still quite limited due to the lack of sufficient isolates. In addition, chemotaxonomy [30], [31] and genome-based taxonomy [32], [33] is of importance to further discriminate the taxonomy of the three strains of *Methanocellales*. Therefore, we decide to propose strain HZ254^T^ as a novel species of the genus *Methanocella*, *Methanocella conradii* sp. nov.

### Description of *Methanocella conradii* sp. nov

*Methanocella conradii* (*con.rad'i.i.* N.L. gen. masc. n. *conradii*, named after Ralf Conrad, who has pioneered the studies on RC-I methanogens in environmental samples). Cells are rods and occur singly with a flagellum. Methane is produced exclusively from H_2_/CO_2_. Acetate is required for growth and yeast extract can stimulate growth. Growth occurs at 37–60°C (optimum 55°C), at pH 6.4–7.2 (optimum 6.8) and with less than 5 g l^−1^ of NaCl (optimum 0–1 g l^−1^). The DNA G+C content is 52.7 mol% determined by genome sequencing. The species was isolated from a rice field soil localized in Hangzhou, China. The type strain is HZ254^T^ (** = **CGMCC 1.5162^T^ **= **JCM 17849^T^ = DSM 24694^T^).

## Supporting Information

Figure S1 **Effects of (a) temperature, (b) pH and (c) NaCl concentration on growth of** ***M. conradii*** **sp. nov.** Specific growth rates at different temperatures were calculated from 2 to 5 replicates by fitting the Gompertz equation [37], the solid line connects the mean values. Effects of pH and NaCl concentration were estimated by following the cumulative methane partial pressures in headspace, data points represent the averages and standard deviations from duplicate or triplicate samples. (**d**) Linear regression of the logarithm of sequential methane partial pressures during exponential growth under the optimal conditions (55°C, pH 6.8, 0 g l^−1^ NaCl), each regression line and equation represents an independent measurement, thus the slope values could be taken as the specific growth rate (*μ* h^−1^) and the doubling time (*G*) was calculated as *G* = ln (2)/*μ*.
(TIF)

Figure S2 **Alignment of near full length of 16S rRNA genes from 12 species.** The numbers after the slash represent the range of the gene length taken for alignment. The alignment was read and printed by Jalview 2.6.1 [38].
(PDF)

Figure S3 **Alignment of deduced McrA amino acid sequences from 12 species.** The numbers after the slash represent the range of the amino acid length taken for alignment. The alignment was read and printed by Jalview 2.6.1 [38].
(PDF)

Table S1 **Assessment of the cultivable consistency of strain HZ254^T^ by successive transfers.**
(DOC)
