# Supplementary figures and images for: Methanocella conradii sp. nov., a Thermophilic, Obligate Hydrogenotrophic Methanogen, Isolated from Chinese Rice Field Soil
Source: PLoS One. 2012 Apr 17;7(4):e35279. doi: 10.1371/journal.pone.0035279 (PMC3328440; doi:10.1371/journal.pone.0035279)

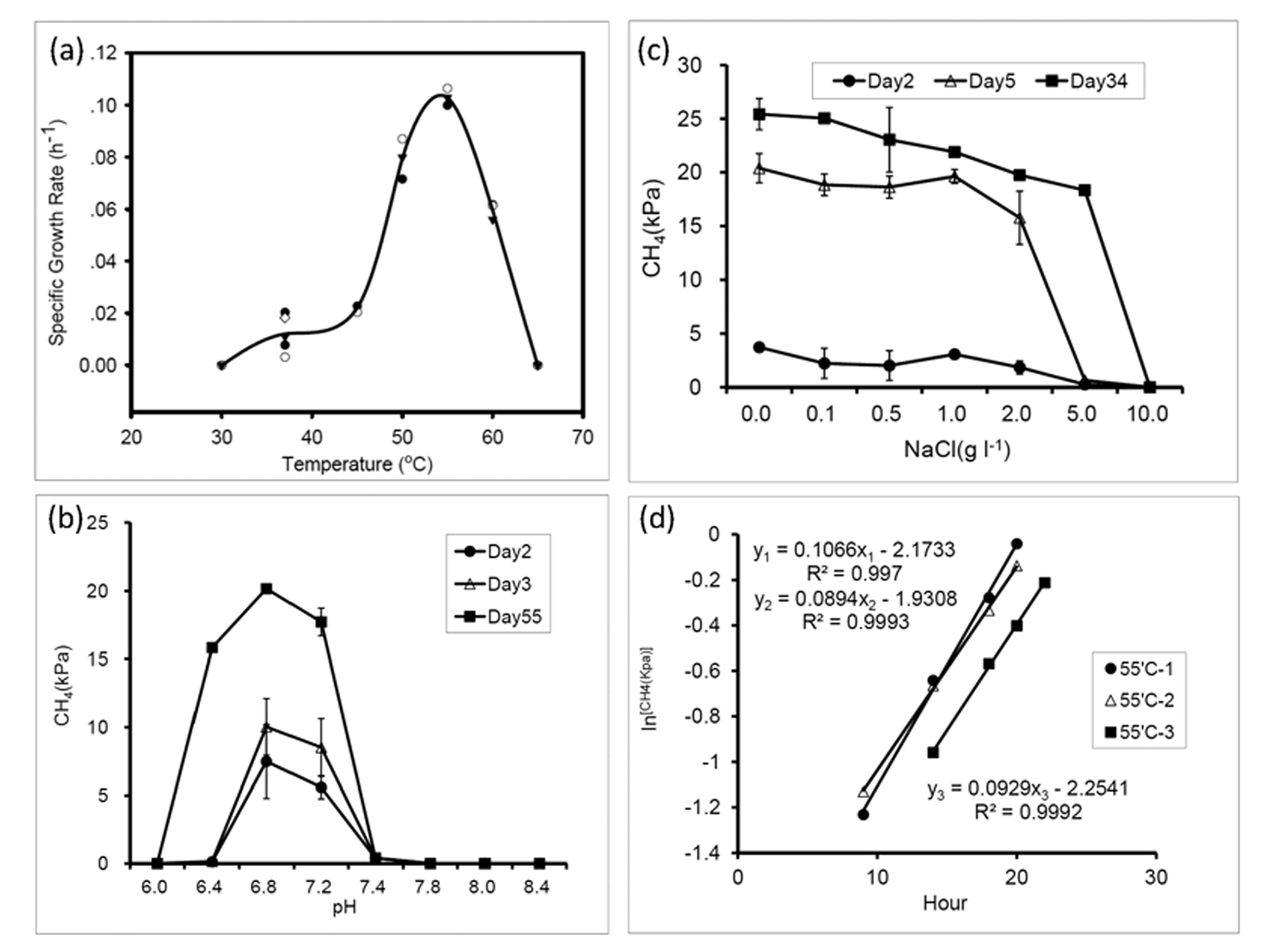

Supplement: Figure S1 — Effects of (a) temperature, (b) pH and (c) NaCl concentration on growth of M. conradii sp. nov. Specific growth rates at different temperatures were calculated from 2 to 5 replicates by fitting the Gompertz equation [37], the solid line connects the mean values. Effects of pH and NaCl concentration were estimated by following the cumulative methane partial pressures in headspace, data points represent the averages and standard deviations from duplicate or triplicate samples. (d) Linear regression of the logarithm of sequential methane partial pressures during exponential growth under the optimal conditions (55°C, pH 6.8, 0 g l−1 NaCl), each regression line and equation represents an independent measurement, thus the slope values could be taken as the specific growth rate (μ h−1) and the doubling time (G) was calculated as G = ln (2)/μ. (TIF) [file pone.0035279.s001.tif]
