# Supplementary material for: Methanocella conradii sp. nov., a Thermophilic, Obligate Hydrogenotrophic Methanogen, Isolated from Chinese Rice Field Soil
Source: PLoS One. 2012 Apr 17;7(4):e35279. doi: 10.1371/journal.pone.0035279 (PMC3328440; doi:10.1371/journal.pone.0035279)
Supplement: Figure S2 — Alignment of near full length of 16S rRNA genes from 12 species. The numbers after the slash represent the range of the gene length taken for alignment. The alignment was read and printed by Jalview 2.6.1 [38]. (PDF) [file pone.0035279.s002.pdf]

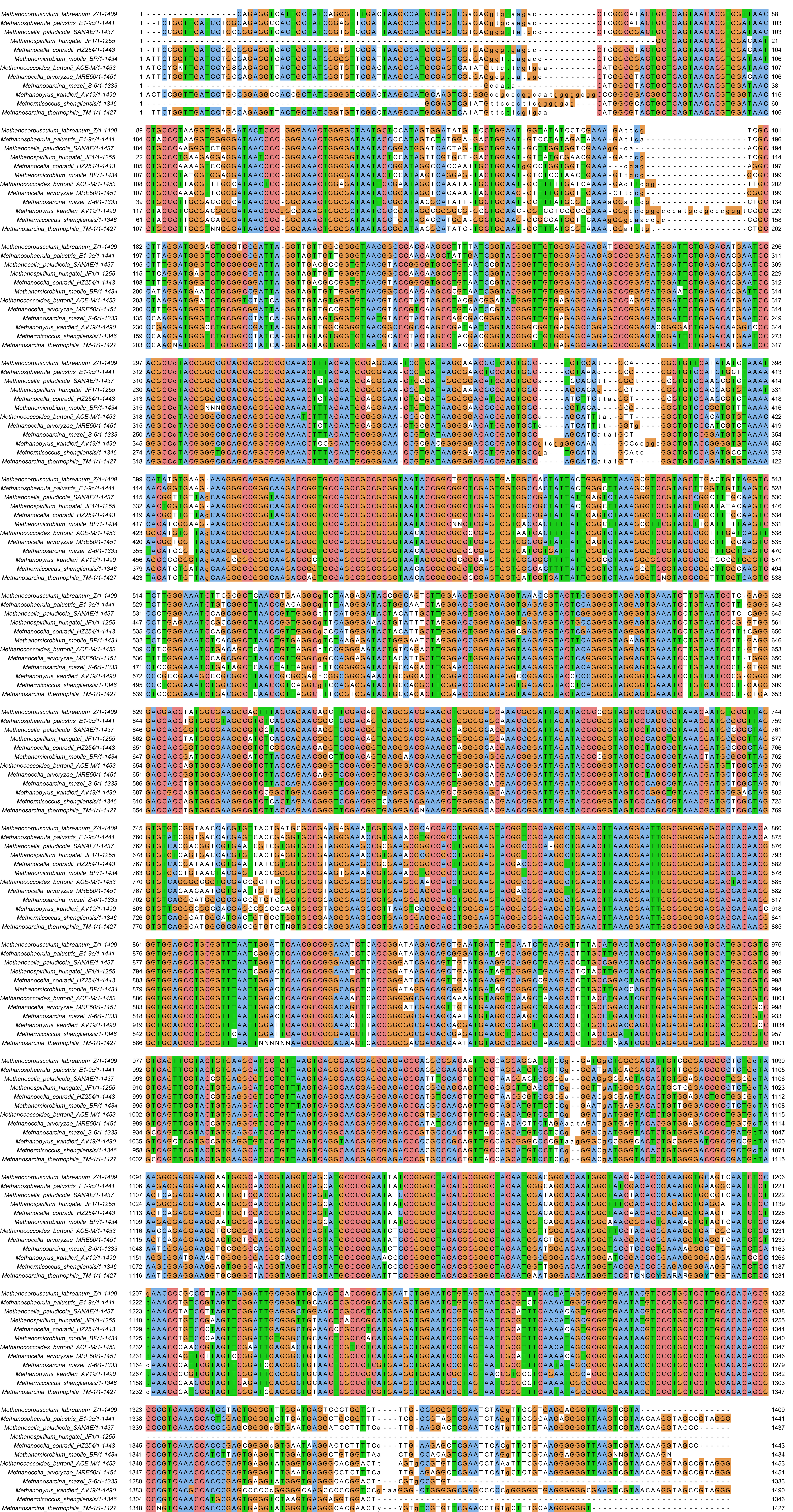
Methanocorpusculum_labreanum_Z/1-1409
Methanosphaerula_palustris_E1-9c/1-1441
Methanocella_paludicola_SANAE/1-1437
Methanospirillum_hungatei_JF1/1-1255
Methanocella_conradii_HZ254/1-1443
Methanomicrobium_mobile_BP/1-1434
Methanococcoides_burtonii_ACE-M/1-1453
Methanocella_arvoryzae_MRE50/1-1451
Methanosarcina_mazei_S-6/1-1333
Methanopyrus_kandleri_AV19/1-1490
Methermicoccus_shengliensis/1-1346
Methanosarcina_thermophila_TM-1/1-1427
