# Supplementary material for: Methanocella conradii sp. nov., a Thermophilic, Obligate Hydrogenotrophic Methanogen, Isolated from Chinese Rice Field Soil
Source: PLoS One. 2012 Apr 17;7(4):e35279. doi: 10.1371/journal.pone.0035279 (PMC3328440; doi:10.1371/journal.pone.0035279)
Supplement: Figure S3 — Alignment of deduced McrA amino acid sequences from 12 species. The numbers after the slash represent the range of the amino acid length taken for alignment. The alignment was read and printed by Jalview 2.6.1 [38]. (PDF) [file pone.0035279.s003.pdf]

```
Methanocorpusculum_labreanum_Z/1-164        1 GSYMSGGVGFTQYATAAYTDNILDDFVYSGMDYIHDKYKVDLKNPNPNDKVKATQEVVNDIATEVNLYGMEQYEQFPTMMEDHFGGSQRAAVLAAASGITTSIATGNSNAGLNGWYLS 118
Methanococcoides_burtonii_ACE-M/1-163       1 GSYMSGGVGFTQYATAAYTNNILDDNLYYNVDYINDKYDGAANKGA-DNKVKATMDVVKDIATESTIYGIENYEKYPTALEDHFGGSQRATVLSAAAGSATALATGNGNAGLSGWYLS 117
Methanosphaerula_palustris_E1-9c/1-155      1 GSYMSGGVGFTQYATAAYTDNILDEFVYYGMDYLKSKYGGYSQAP-------ATQEVVNDLATEVTLNAMEQYEQFPTMMEDHFGGSQRASVIAAASGLTTSIGTGNSNAGLNGWYLS 111
Methanomicrobium_mobile_BP/1-146            1 -----------------YTDNILDEFTYYGMDYIKDKYGVDYKNPSPAKLVKPTQEVVNDIATEVNLNGMEQYEQYPTMMEDHFGGSQRASVLAASCGITTSIATGNSNAGLNAWYLS 101
Methermicoccus_shengliensis_ZC-1/1-144      1 GSYMSGGVGFTQYATAAYTNNILDDFSYYGYEYAVDKYGGP-------AQAPATLETVKDIATETAIYAIEQYEYFPTLLEDQFGGSQRAAVVAAAAGIATGLATGNSQAGLSGWYLA 111
Methanosarcina_mazei_S-6/1-160              1 GSYMSGGVGFTQYATAAYTDDILDNNTYYDVDYINDKYNGAANLGT-DNKVKATLDVVKDIATESTLYGIETYEKFPTALEDHFGGSQRATVLAAASGVACALATGNANAGLSGWYLS 117
Methanosarcina_thermophila_TM-1/1-163       1 GSYMSGGVGFTQYATAAYTDDILDNNTYYNVDYINDKYNGAAKVGK-DNKIKATLEVVKDIATESTIYGIETYEKFPTALEDHFGGSQRATVLAAAAGVATALATGNANAGLSGWYLS 117
Methanospirillum_hungatei_JF-1/1-162        1 GSYMSGGVGFTQYATAAYTDNILDEFTYYGMDYIKDKYKVDWKNPSPKDKVKPTQEIVNDIAGEVTLNAMEQYEQFPTMMEDHFGGSQRAGVIAAASGLSVGVATANSNAGLNGWYLS 118
Methanopyrus_kandleri_AV19/1-156            1 GSYMSGGVGFTQYATAVYPDNILDDYVYYGLEYVEDKYGIA--------EAEPSMDVVKDVATEVTLYGLEQYERYPAAMETHFGGSQRAAVCAAAAGCSTAFATGHAQAGLNGWYLS 110
Methanocella_paludicola_SANAE/1-147         1 GSYMSGGVGFTQYATAAYTDDILDDFTYYGYDYAKGKYKIG--------QTKPTMDIVNDLSTEVTLYGIEQYEKYPTTLEDHFGGSQRATVLSAAAGVTTAIATGNSNAGLSGWYMS 110
Methanocella_arvoryzae_MRE50/1-152         1 ----?GGVGFTQYATAAYTDDILDDFCYYGYDYIKGKYGIA--------KAKPTMDVVNDIGTEVTLYGIEQYEKYPTTLEDHFGGSQRATVLSAAAGVTTSLATGNANAGLSAWYLS 106
Methanocella_conradii_HZ254/1-155           1 GSYMSGGVGFTQYATAAYTDDILDDFTYYGYDYAKGKYKM---------PTKPTMDIVNDLGTEVTLYGIEQYEKYPTTLEDHFGGSQRATVLAAASGVTTALATGSSNAGLSAWYLS 109

Methanocorpusculum_labreanum_Z/1-164      119 MLLHKDGWSRLGFFGYDLQDQCGSANSLSIRPDEGCIGEFRGPNYP 164
Methanococcoides_burtonii_ACE-M/1-163     118 MYLHKEALGRLGFFGFDLQDQCGATNVFSFQSDEGLPLELRGPNYP 163
Methanosphaerula_palustris_E1-9c/1-155    112 MLMHKEGWSRLGFFGYDLQDQCGSANSMSIRPDEGLLGELRGPN-- 155
Methanomicrobium_mobile_BP/1-146          102 MLMHKGGWSRLGFFGYDLQDQCGSANSLSMEPDRGLIGELRGPNY- 146
Methermicoccus_shengliensis_ZC-1/1-144    112 QYLLKEAEGRLGFFGYDLQDQCGAANVFSYQSD------------- 144
Methanosarcina_mazei_S-6/1-160            118 MYVHKEAWGRLGFFGFDLQDQCGATNVLSYQGDEGLPDELRGP--- 160
Methanosarcina_thermophila_TM-1/1-163     118 MYLHKEAWGRLGFFGYDLQDQCGATNVLSYQGDEGLPNELRGPNYP 163
Methanospirillum_hungatei_JF-1/1-162      119 MLMHKEGWSRLGFFGYDLQDQCGSTNSLSVRPDEGCIGEYRGP?-- 162
Methanopyrus_kandleri_AV19/1-156          111 QILHKEGQGRLGFYGYALQDQCGAANSLSVRSDEGLPLELRGPNYP 156
Methanocella_paludicola_SANAE/1-147       111 MYLHKEAWGRLGFFGYDLQDQCGATNVFSCRSDEGAI--------- 147
Methanocella_arvoryzae_MRE50/1-152        107 MYLHKEAWGRLGFFGYDLQDQCGATNVFSCRSDEGAIDELRGPNYP 152
Methanocella_conradii_HZ254/1-155         110 MYLHKEAWGRLGFFGYDLQDQCGATNVFSCRSDEGAMCELRGPNYP 155
```
