# Supplementary material for: Methanocella conradii sp. nov., a Thermophilic, Obligate Hydrogenotrophic Methanogen, Isolated from Chinese Rice Field Soil
Source: PLoS One. 2012 Apr 17;7(4):e35279. doi: 10.1371/journal.pone.0035279 (PMC3328440; doi:10.1371/journal.pone.0035279)
Supplement: Table S1 — Assessment of the cultivable consistency of strain HZ254T by successive transfers. (DOC) [file pone.0035279.s004.doc]

| Transfers | Subculture date | Storage date | Growth time (day) | Storage time (day) |
| --- | --- | --- | --- | --- |
| 1th | 2009/7/3 | 2009/7/17 | 14 | 4 |
| 2nd | 2009/7/21 | 2009/7/31 | 10 | 30 |
| 3rd | 2009/8/21 | 2009/9/6 | 16 | 19 |
| 4th | 2009/9/25 | 2009/10/5 | 10 | 148 |
| 5th | 2010/3/2 | 2010/3/12 | 10 | 502 |
| 6th | 2011/7/27 | 2011/9/8 | 43 | 64 |
| 7th | 2011/11/11 | 2011/11/21 | 10 | N.A. |

Transfers: successive transfers of a starting culture from 2009/7/3.

Subculture date: the inoculation date of each transfer.

Storage date: the date that each fully grown subculture was kept at 4°C.

Growth time: the time that each subculture took to achieve full growth.

Storage time: the time that each subculture was kept at 4°C until its next transfer.

N.A.: not applicable, as no further transfer was operated.

Note: (1) the growth of the cultures was routinely followed by measuring methane production, microscopic analysis or visual observation of turbidity. (2) A fully grown culture denoted that the culture had approximately grown to the late-logarithmic phase. (3) Normally two or more replicates were subjected to subculture, cultures in this table was thus just one set of examples to document the cultivable consistency of the strain.
